# Long-term outcome of renal nerve denervation (RDN) for resistant hypertension

**DOI:** 10.1038/s41440-022-00910-7

**Published:** 2022-04-07

**Authors:** Pariya Panchavinnin, Sirisawat Wanthong, Weranuj Roubsanthisuk, Damras Tresukosol, Peera Buranakitjaroen, Chavalit Chotruangnapa, Wattana Watanapa, Rungtiwa Pongakasira, Nattawut Wongpraparut

**Affiliations:** 1 Division of Cardiology, Department of Medicine, Faculty of Medicine Siriraj Hospital, Bangkok, Thailand; 2 Division of Hypertension, Department of Medicine, Faculty of Medicine Siriraj Hospital, Bangkok, Thailand; 3 Department of Physiology, Faculty of Medicine Siriraj Hospital, Bangkok, Thailand; 4 Her Majesty’s Cardiac Center, Faculty of Medicine Siriraj Hospital, Bangkok, Thailand

**Keywords:** Resistant hypertension, Renal nerve denervation, Long-term outcome

## Abstract

We aimed to determine the long-term outcome of renal denervation (RDN). All patients with resistant hypertension who underwent RDN between 2012 and 2018 at Siriraj Hospital were included in the study. Patients were followed up at 3, 6, and 12 months and then annually up to 9 years. Effectiveness of the RDN outcome was defined by either (1) a reduction in office systolic BP ≥ 10 mmHg, (2) a reduction in the number of antihypertensive drugs taken, or (3) both outcomes being achieved. In total, 18 RDN procedures were performed during the study period. The mean and longest follow-up periods were 52 months and 104 months, respectively. Heterogeneous BP responses after RDN for resistant hypertension were observed. Effectiveness of the RDN outcome was achieved in 88% of the patients at 1 year and in >80% of the patients during the entire follow-up at each time point up to 9 years.

## Abstract (web summary)

Heterogenous BP responses after RDN for resistant hypertension. The bar graph represents the difference in SBP at 6 months after RDN compared with the baseline SBP, for each patient (1A). Efficacy of the renal denervation procedure. Percentage of patients whose SBP decreased ≥10 mmHg from baseline or whose medication decreased from baseline (1B).

## Graphical abstract

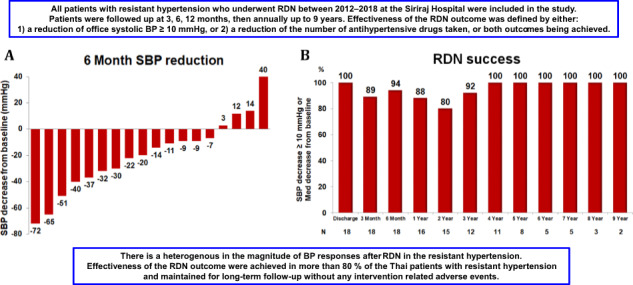

## Introduction

Resistant hypertension is defined as seated office blood pressure (BP) > 140/90 mmHg in a patient treated with three or more antihypertensive medications at optimal (or maximal tolerated) doses, including a diuretic, and after excluding pseudoresistance as well as drug-induced hypertension and secondary hypertension [1]. Initially, our case series of resistant hypertension patients who underwent renal denervation (RDN) showed sustained BP reduction at 6 months [2]. Asian patients with resistant hypertension are a target population of RDN treatment [3, 4]. Doubts were cast on its efficacy when the SYMPLICITY HTN-3 study did not show a statistically significant BP reduction in RDN compared to sham treatment [5]. SPYRAL HTN-OFF MED and SPYRAL HTN-ON MED reported significant systolic BP reductions of more than 5 mmHg in the RDN groups regardless of whether hypertensive drug naïve or persistent patients were involved [6, 7]. However, the patients in those trials were patients with mild to moderate hypertension. Data on the long-term efficacy of RDN in resistant hypertension remain lacking. Consequently, we aimed to assess the long-term efficacy of RDN in resistant hypertension.

Point of view **Clinical relevance**

Effectiveness of the RDN outcome was achieved in >80% of the patients with resistant hypertension and was maintained over a long-term follow-up without any intervention-related adverse events.

**Future direction**

A long-term study to assess the effect of RDN on cardiovascular outcomes is warranted.

**Consideration for the Asian population.**

RDN should be considered as an adjunct treatment in addition to antihypertensive therapy in Asian patients with resistant hypertension.

## Methods

The Siriraj Institutional Review Board (SIRB) of the Faculty of Medicine Siriraj Hospital, Mahidol University approved this study (COA no. Si 252/2013), and the study protocol conforms to the ethical guidelines of the 1975 Declaration of Helsinki. All of the resistant hypertension patients who underwent percutaneous renal nerve denervation at our institution between 2012 and 2018 were included in the study. The patients’ baseline demographics were recorded. The number and dose of antihypertensive medications before and after RDN were recorded. Office BP measurement was performed while patients sat with their arms resting on the table with the mid-arm at heart level and the back supported on the chair in a quiet room and relaxed for 5 min. Smoking and caffeine were avoided. The BP cuff was sized according to the individual arm cuff. Three BP measurements were performed and calculated based on the average of the last 2 measurements. Procedural data, including the procedure time, premedication, renal angiography data, and area and number of radiofrequency ablations, were recorded. RDN was performed according to a standard protocol. Patients were followed up at 3 months and 6 months after the procedure and then annually up to 9 years.

## Results

Between 2012 and 2018, 17 patients who underwent 18 RDN procedures were included in the study, and their records were reviewed. The patients had a mean systolic BP of 168.6 ± 22.7 mmHg despite taking 5.2 ± 1.2 antihypertensive drugs. The baseline clinical characteristics are shown in Table 1. The prevalence of accessory renal arteries in this study was 11.8% (2 patients). The first 7 patients (38.9%) underwent RDN using a Symplicity catheter^®^, while the latter 11 patients (61.1%) used a Symplicity Spyral catheter^®^. The median RDN time was 39 min (IQR 25–62 min), with a median total catheter lab time of 64 min (46–85 min). There was a median of 20 (IQR 16–24) total successful ablations performed. RF ablation was performed at the distal branch in 11 patients (61.1%). One patient developed renal artery vasospasm, which was successfully treated with IA nitroglycerin. There were no long-term complications related to the procedures.

**Table 1:** Baseline clinical characteristics, co-morbid, blood pressure measurement, and number and dose of antihypertensive drugs

Variables	*N* = 18
Age (years)	61.1 ± 17.7
Female	12 (66.7)
BMI (kg/m^2^)	29.3 ± 5.3
eGFR (ml/minute/1.73 m^2^)	59.4 (35.8,80.4)
eGFR ≤ 60 ml/min/1.73 m^2^	9 (50.0)
OSA	3 (16.7)
Stroke and TIA^a^	4 (22.2)
DM	10 (55.6)
CAD^a^	9 (50.0)
SBP (mmHg)	168.6 ± 22.7
DBP (mmHg)	81.1 ± 21.4
HR (bpm)	87.3 ± 19.9
Pre-RDN ABPM (*n* = 9)	
24 h SBP (mmHg)	170.50 ± 38.58
24 h DBP (mmHg)	89.25 ± 41.12
24 h HR (bpm)	90.25 ± 28.06
Daytime SBP (mmHg)	169.25 ± 43.95
Daytime DBP (mmHg)	90.50 ± 44.38
Daytime HR (bpm)	90.25 ± 27.44
Nighttime SBP (mmHg)	176.25 ± 23.34
Nighttime DBP (mmHg)	89.25 ± 37.92
Nighttime HR (bpm)	81.00 ± 18.71
Number of antihypertensive drugs	5.2 ± 1.2
ACEI/ARB	16 (88.9)
Beta- blocker	15 (83.3)
CCB	16 (88.9)
Diuretic group	14 (77.8)
Aldosterone antagonist	4 (22.2)
Alpha-blockers	12 (66.7)

Data presented as number and percentage, mean ± standard deviation, or median (IQR)

*BMI* body mass index, *eGFR* estimated glomerular filtration rate, *OSA* obstructive sleep apnea, *TIA* transient ischemic attack, *DM* diabetes mellitus, *CAD* coronary artery disease, *SBP* systolic blood pressure, *DBP* diastolic blood pressure, *HR* heart rate, *ABPM* ambulatory blood pressure monitoring, *CCB* calcium-channel blockers, *ACEI* angiotensin converting enzyme inhibitors, *ARB* angiotensin II receptor blockers

^a^These events occurred >12 months before the procedure

### Long-term outcomes

Of all the cases, the longest follow-up was 104 months, with a mean follow-up period of 52.39 ± 30.96 months. The reduction in systolic BP was greater immediately after the procedure, with a mean change in systolic BP of −31.9 ± 17.3 mmHg at the date of discharge, −24.3 ± 32.0 mmHg at 6 months, −16.8 ± 32.3 mmHg at 1 year, −15.3 ± 29.4 mmHg at 3 years, −15.3 ± 35.2 at 5 years, −14.8 ± 27.9 at 7 years, and −30.0 ± 12.7 at 9 years. The average antihypertensive drugs received by the patients decreased from 5.2 ± 1.2 at baseline to 4.7 ± 1.3, 3.6 ± 1.6, 3.7 ± 1.7, and 4.1 ± 1.6 at the date of discharge, 6-month, 1-year, and 2-year follow-ups, respectively, and were sustained at 4 drugs after the 2-year follow-up. Three patients had noncardiac death during the follow-up period.

There is a heterogeneity in the magnitude of BP responses after RDN in resistant hypertension. The mean systolic BP reduction was −24.3 ± 32.0 mmHg at 6 months, but the range of the systolic BP difference between baseline and 6 months was −72 mmHg to +40 mmHg. The heterogeneous BP responses after RDN in resistant hypertension are demonstrated in Graphical Abstract Fig. 1A. This outcome could be partly affected by the adjustment of antihypertensive medication. We assessed the effectiveness of the renal denervation procedure in achieving a reduction in systolic BP of ≥10 mmHg, a reduction in the number of antihypertensive drugs or both, as shown in Graphical Abstract Fig. 1B. Effectiveness of the RDN outcome was achieved in >80% of the patients during the entire follow-up at each time point.

### Repeated renal denervation

One patient in our study underwent a second RDN due to a resurgence of BP after her first RDN. Her office blood pressure was raised to 184/110 mmHg and 196/113 mmHg at the fourth and fifth years of follow-up, respectively. After her second RDN, her BP dramatically decreased to 102/68 mmHg. Furthermore, she was also able to decrease her antihypertensive drugs taken from 5 drugs to 2 drugs.

## Discussion

The present study investigated the long-term effectiveness of RDN outcomes in patients with resistant hypertension. The main findings of the study were as follows: (1) there was heterogeneity in the magnitude of BP reduction from baseline at the 6-month follow-up after RDN; (2) effectiveness of the RDN outcome was achieved in >80% of the patients during the entire follow-up at each time point up to 9 years; and (3) rebound hypertension occurred in 1 patient but was then successfully treated with re-do RDN.

This study provided long-term data on the effective outcome of RDN for resistant hypertension, and this effectiveness was sustained for up to 9 years without any intervention-related adverse events. The GSR reported the 3-year outcome after RDN. Systolic BP reduction after RDN was sustained over 3 years, with office systolic BP (−16.5 ± 28.6 mmHg) [8] and (−32 ± 18.8 mmHg) in GSR Korea [9]. The findings of a greater effect of RDN on long-term BP reduction in Asia were consistent with a prior report by Kario et al. [10].

Effectiveness of the RDN outcome was achieved in >80% of the patients during the entire follow-up at each time point. The heterogeneous BP responses after RDN were mainly from appropriate patient selection. Patient preference should also be part of the decision-making in patient selection [11]. The ideal candidates for RDN are patients with autonomic hypersensitivity. Several studies have shown the benefit of autonomic function in guided selection of patients who are good responders to RDN. The use of cardiac baroreflex sensitivity (BRS), heart rate variability (HRV) and baseline 24-hour HR > 73.5 beats per minute (BPM) in predicting good responders to RDN was proposed [12–14]. From our ambulatory BP monitoring (ABPM) data, all of the patients with baseline pre-RDN 24-hour HR > 73.5 BPM exhibited effectiveness of the RDN outcome at 6 months. Esler et al. suggested the clinical characteristics of patients with neurogenic hypertension, such as hypertensive patients with obesity, obstructive sleep apnea (OSA), isolated systolic hypertension in the young, or a high ambulatory HR [13]. Other clinical clues of sympathetic hypersensitivity, such as OSA and atrial fibrillation, could also help identify patients. All three patients with OSA in our study were good responders after RDN.

Rebound hypertension occurred in 1 patient. This patient had undergone a second RDN because of a resurgence of BP after the fourth year. Booth et al. found reinnervation 11 months after denervation in an animal study [15]. The successful reduction of BP in the repeated RDN of our case was similar to the results from recent reports/case series.

In terms of limitations, this study was a registry. Blood pressure assessment was office blood pressure, not 24-hour ABPM.

## Asian perspectives

Ethnic differences in hypertension characteristics, hypertension-related cardiovascular disease (CVD) and BP reduction after RDN were reported between Asian and other ethnicities. Stroke occurs more frequently than myocardial infarction in Asian countries compared to Western countries [16]. Asians have more prevalent masked hypertension, which is associated with increased CVD risks [17]. Moreover, greater BP reduction after RDN in Asian versus non-Asian patients was observed [10]. This study provides long-term data on RDN effectiveness. Further research should be conducted to assess mortality and CVD outcomes after RDN in Asians versus other ethnicities.

## Conclusion

There is heterogeneity in the magnitude of BP responses after RDN in resistant hypertension. Effectiveness of the RDN outcome was achieved in >80% of the Thai patients with resistant hypertension and was maintained over a long-term follow-up without any intervention-related adverse events.
